# A computational modeling method for root canal endoscopy using a specific CBCT filter: A new era in the metaverse of endodontics begins

**DOI:** 10.1590/0103-6440202205078

**Published:** 2022-08-26

**Authors:** Mike R Bueno, Carlos Estrela

**Affiliations:** 1 Professor of Radiology, CROIF, Diagnostic Imaging Center, Cuiabá, Brazil; 2 Professor of Endodontics, Department of Stomatology Sciences, Federal University of Goiás, Goiânia, Brazil

**Keywords:** Cone-beam computed tomography, endoscopy, e-Vol DX, metaverse, root canal endoscopy, software

## Abstract

A contemporary technological revolution has started a new era in the metaverse of Endodontics, a world of virtual operational possibilities that use an exact replica of the natural structures of the maxillofacial complex. This study describes a modeling method for root canal endoscopy using modern cone-beam CT (CBCT) software in a series of clinical cases. The method consists in acquiring thin CBCT slices (0.10mm) in the coronal, sagittal, and axial planes. A specific 3D volume filter, the pulp cavity filter of the e-Vol DX CBCT software, was used to navigate anatomical root canal microstructures, and to scan them using root canal endoscopy. The pulp cavity filter should be set to synchronize CBCT scans from 2D mode - multiplanar reformations (MPR) - to 3D mode - volumetric reconstruction. This filter, when adopting the option of volumetric reconstruction, the developed algorithm leaves the dentin density in transparent mode so that the pulp cavity may be visualized. The algorithm applied performs the suppression (visual) of areas with dentin density. This ensures 3D visualization of the slices and the microanatomy of the root canal, as well as a dynamic navigation throughout the pulp cavity. This computational modeling method adds new resources to Endodontics, which may impact the predictability of root canal treatments positively. The virtual visualization of the internal anatomy of an exact replica of the canal ensures better communications, reliability, and clinical operationalization. Root canal endoscopy using this novel CBCT filter may be used for clinical applications together with innovative digital and virtual-reality resources that will be naturally incorporated into the principles of Endodontics.

## Resumo

Uma revolução tecnológica contemporânea deu início a uma nova era no metaverso da Endodontia, um mundo de possibilidades operacionais virtuais que utilizam uma réplica exata das estruturas naturais do complexo dentomaxilofacial. Este estudo descreve um método de modelagem computacional para a endoscopia do canal radicular, usando um moderno software de tomografia computadorizada de feixe cônico (TCFC), em uma série de casos clínicos. O método consiste na aquisição de finos *slices* de TCFC (0,10mm) nos planos coronal, sagital e axial. Um filtro específico de TCFC (filtro cavidade pulpar do software e-Vol DX) foi usado para navegar nas microestruturas anatômicas do canal radicular, e escanear para a aplicação da endoscopia do canal radicular. Este filtro foi configurado para sincronizar as imagens de TCFC em modo 2D - reformações multiplanares (MPR) para o modo 3D - reconstrução volumétrica. O filtro *Pulp Cavity* ao adotar a opção de reconstrução volumétrica, um algoritmo desenvolvido deixa a densidade dentinária em modo transparente, para que a cavidade pulpar possa ser melhor visualizada. O algoritmo aplicado realiza a supressão (visual) das áreas com densidade dentinária. Este modo de aplicação garante a visualização 3D da microanatomia do canal radicular, bem como permite uma navegação dinâmica por toda a cavidade pulpar. O método de modelagem computacional agrega novos recursos à Endodontia, o que pode impactar positivamente na previsibilidade dos tratamentos endodônticos. A visualização virtual da anatomia interna de uma réplica exata do canal radicular garante melhor comunicação, confiabilidade e operacionalização clínica. O exame de endoscopia do canal radicular com este novo filtro (Pulp cavity) pode ser usada para aplicações clínicas juntamente com recursos digitais e de realidade virtual inovadores que serão naturalmente incorporados aos princípios da Endodontia.

## Introduction

The clinical management of root canal treatments (RCT) requires an in-depth knowledge of internal tooth anatomy and biological factors involved in pulp and periapical inflammation and infection, as well as of cleaning and shaping strategies and immune responses of the host. The advancement and mastery of scientific and technological knowledge have ensured important therapeutic advances in clinical practice ^1^ ^,^ ^2^ ^,^ ^3^ ^,^ ^4^.

The internal tooth anatomy, in particular, may hold secrets not informed by conventional imaging studies, such as periapical radiographs. These images provide two-dimensional views of multidimensional structures and, therefore, omit many anatomical details that may be essential for a successful clinical outcome of RCT ^5^ ^,^ ^6^. Cone-beam CT (CBCT) provides more accurate and detailed information about the anatomical structures of a root canal system, which has a positive impact on RCT outcomes and improves predictability. CBCT imaging studies show the anatomic architecture of a tooth, which includes its root canals, apical foramina, and isthmuses, as well as the canal shape and curvature. This information is an important contribution to RCT planning and clinical decision-making ^6^ ^,^ ^7^ ^,^ ^8^ ^,^ ^9^ ^,^ ^10^.

Endoscopy has addressed the need to see inside the human body, especially inside structures and organs ^11^. More than two hundred years ago, Philipp Bozzini introduced the idea of lighting the interior of human body cavities to examine the inside of internal organs and invented his Lichtleider, or light-conductor, today recognized as the first endoscope ^11^. Since then, endoscopy has been responsible for significant improvements and changes in both clinical directions and decision-making and has replaced several clinical procedures ^12^ ^,^ ^13^ ^,^ ^14^.

Endoscopic improvements influenced other associated areas of human knowledge, such as optics, mechanics, photography, video, and imaging studies ^12^ ^,^ ^13^ ^,^ ^14^ ^,^ ^15^ ^,^ ^16^ ^,^ ^17^. New technologies that provide information about anatomical and pathological characteristics have contributed to the improvement of clinical decision-making and RCT predictability. Some of these technologies are CBCT scans ^6^ ^,^ ^7^ ^,^ ^8^ ^,^ ^9^ ^,^ ^10^, operating microscopes and other magnification techniques, ultrasound scanning, and optical coherence tomography ^6^ ^,^ ^7^ ^,^ ^8^ ^,^ ^9^ ^,^ ^10^ ^,^ ^11^ ^,^ ^12^ ^,^ ^13^ ^,^ ^14^ ^,^ ^15^ ^,^ ^16^ ^,^ ^17^ ^,^ ^18^ ^,^ ^19^ ^,^ ^20^ ^,^ ^21^ ^,^ ^22^ ^,^ ^23^ ^,^ ^24^ ^,^ ^25^ ^,^ ^26^ ^,^ ^27^ ^,^ ^28^ ^,^ ^29^ ^,^ ^30^, ^31^, ^32^.

Significant advances with the advent and incorporation of new technologies in the health area have allowed expanding the process of diagnosis and treatment of various diseases with a profound influence on the quality of life. The improvement with the exploration of the metaverse in the health area may impact not only on clinical decision-making, but on a greater reliability in the therapeutic clinical management for the professional, as well as in the realistic forms of teaching and practical application at a distance. More specifically in Dentistry, the use of sophisticated CBCT software ^10^ associated with artificial intelligence is one of the advances incorporated into Endodontics. Locurcio ^33^ ^)^ analyzed the future of Dentistry and dental education in the metaverse and found that the metaverse is an extension of the internet in which users interact with each other and the environment around them using virtual and augmented reality resources. Kurian et al. ^34^ discussed the exploration of the multiverse and the future possibility of performing a RCT with radiographs or 3D images of the morphology of the canal and a view of its anatomical extent.

The development of a novel CBCT software has resulted in improvements in image sharpness and, consequently, in the visualization of root canal morphology, with a great increase in RCT quality ^10^. CBCT cinematic rendering for clinical decision-making has been recently introduced in Endodontics ^9^. Cinematic rendering is a 3D reconstruction technique that generates more photorealistic 3D images from CBCT scans, with a high-quality resolution and a refined final image ^8^ ^,^ ^9^ ^,^ ^10^.

The current understanding of the characteristics of early Endodontics in the metaverse and the concept of looking inside the root canal brings a new realistic, virtual and applicable perspective to clinical practice. This study discusses the development of a modeling method for root canal endoscopy using a new CBCT filter within the framework of cinematic rendering.

## Material and Methods

This modeling method is described in a series of four clinical cases treated with the aid of a novel filter developed to work with the e-Vol DX CBCT software (CDT Software; São José dos Campos, Brazil) and a cinematic reading tool that uses AI-related algorithms. The modeling method of root canal configuration includes root canal endoscopy and internal scanning with a specific filter - the pulp cavity filter - of the e-Vol DX CBCT software.

The CBCT scans were selected from a database of patients referred to a private radiology service (CROIF, Cuiabá, MT). They had different pulpal and periapical diagnoses. In all clinical endodontic cases described here, CBCT scans were acquired using a PreXion 3D scanner (PreXion 3D Inc., San Mateo, CA) and a high-resolution standard protocol: field of view - 5.6 cm, voxel - 0.108 mm, exposure time - 37 seconds (16 bits), tube voltage - 90 kVp, tube current - 4 mA, and thickness (isotropic voxel) - 0.100mm. CBCT scans were reconstructed using the proprietary software native to the scanner. The volume was exported as an axial multi-DICOM series and imported into the e-Vol DX software running on a PC workstation equipped with an Intel i7-7700K processor, 4.20 GHz (Intel Corp., Santa Clara, CA), NVIDIA GeForce GTX 1070 video card (NVIDIA Corporation, Santa Clara, CA), Dell P2719H monitor at a resolution of 1920X1080 pixels (Dell Technologies Inc., Round Rock, TX) and Windows 10 Pro (Microsoft Corp., Redmond, WA). The original CBCT scans were visualized using the scanner’s original software. The volume was exported as a multi-file DICOM dataset and reconstructed using the e-Vol DX software.

### Computational Modeling method for root canal endoscopy

A modeling method for root canal endoscopy was proposed to view and navigate within the root canal anatomical microstructures, which may not be possible when using conventional imaging exams, such as periapical radiographs. Professionals may find a greater amount of information once they have mastered and become familiar with the software.

**Figure 1:**
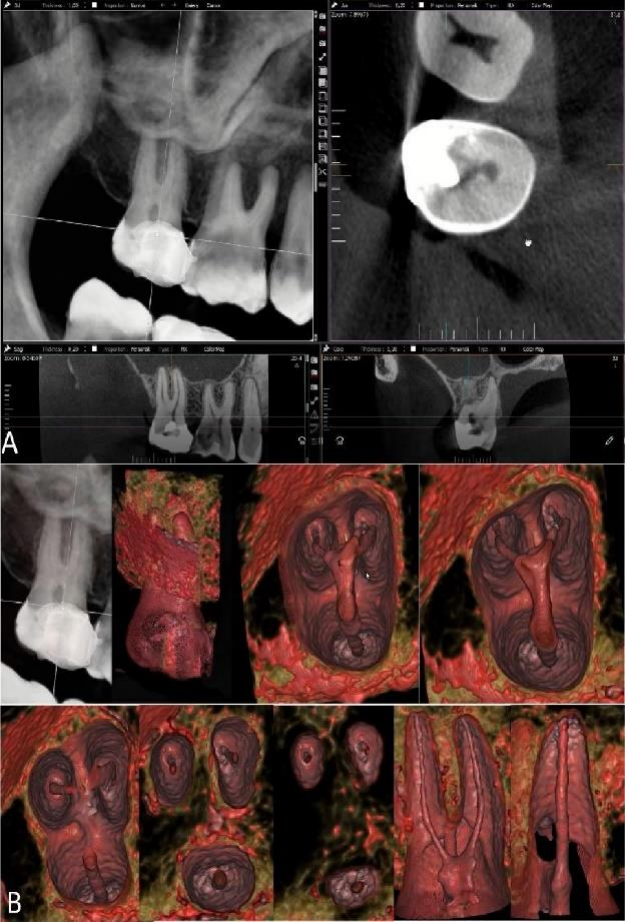
CBCT scan of the maxillary right second molar in 2D MPR (A) and 3D cinematic rendering (B) showed the coronal chamber and root canals and revealed the characteristic anatomical shape of each root, the presence of a lateral canal in the palatal root and the position of the apical foramen.

**Figure 2:**
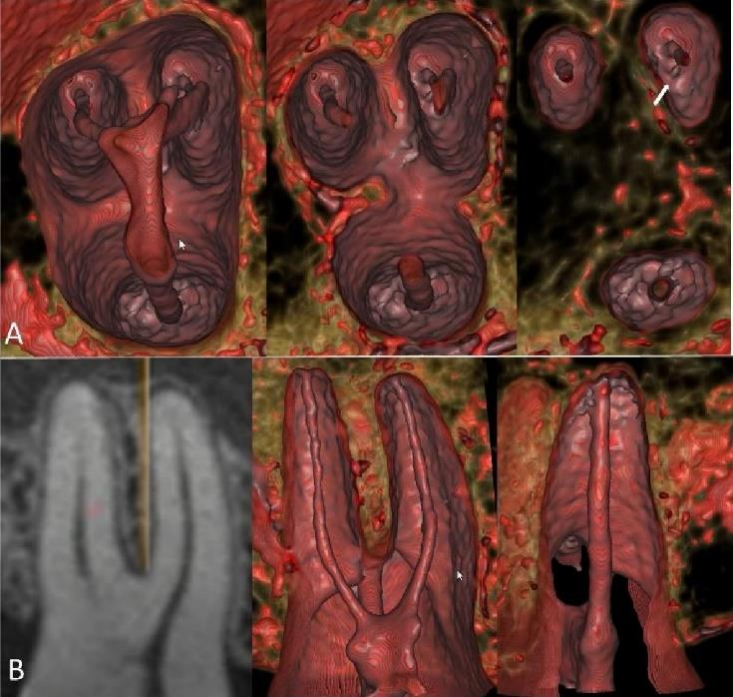
3D cinematic rendering with higher magnification and more details of Figure 1, showing anatomical aspects of the pulp cavity in the root thirds (A) and a panoramic view in 2D MPR and 3D cinematic rendering of the pulp cavity from the buccal and palatine canals (B).

**Figure 3:**
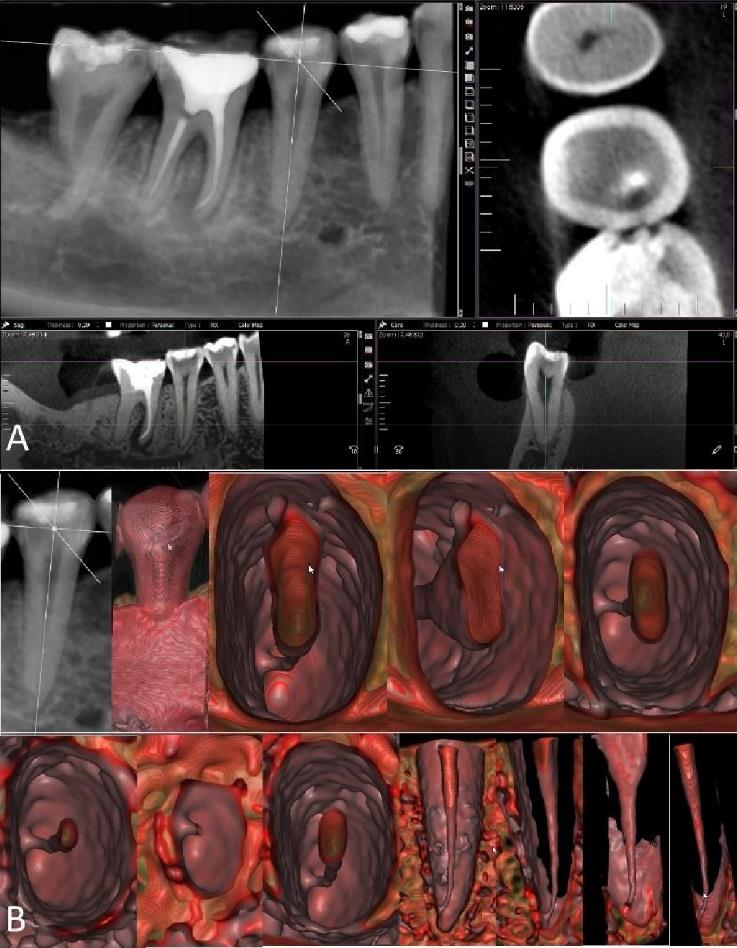
CBCT scan of the mandibular right second premolar in 2D MPR (A) and 3D cinematic rendering (B) showing the nuances of the pulp cavity and the lateral position of the apical foramen terminus.

**Figure 4:**
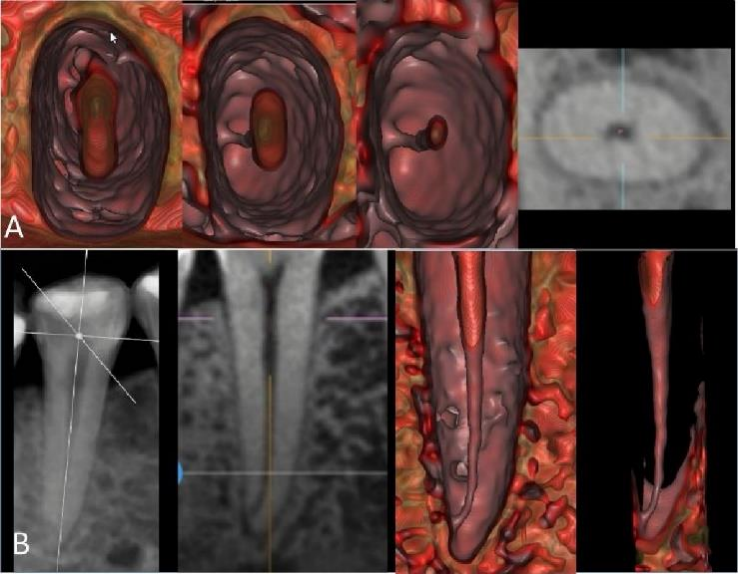
Higher magnification and more details of Figure 3, visualizing the pulp cavity in different root thirds, and identifying the lateral and distal position of the apical foramen below the radiographic apex.

**Figure 5:**
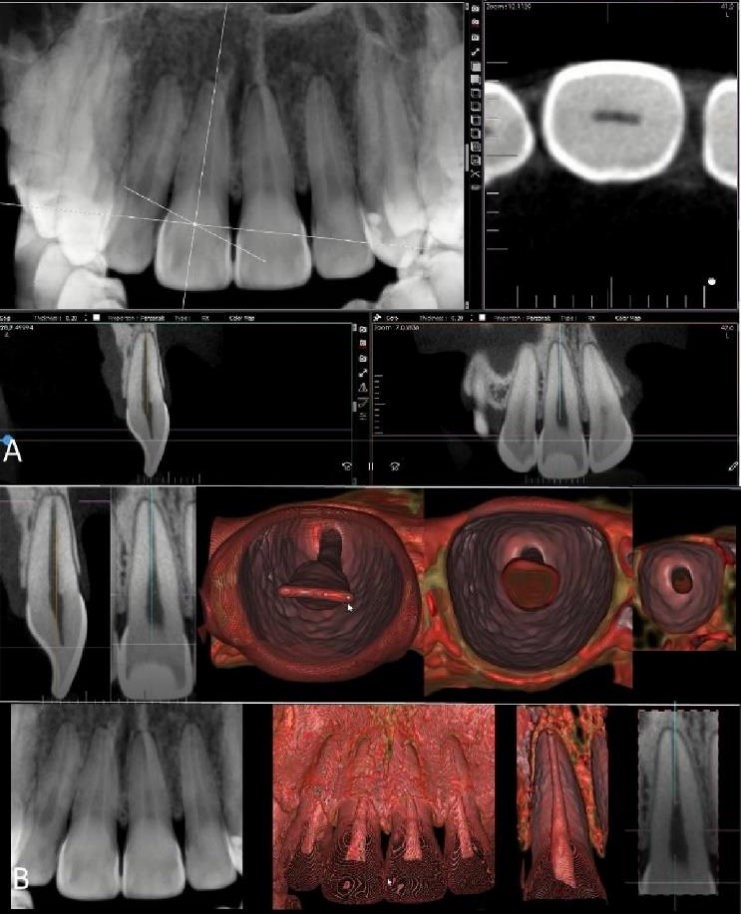
CBCT scan of the maxillary right central incisor in 2D MPR (A) and 3D cinematic rendering (B). The multidimensional internal navigation showed the pulp cavity and the central position of the apical foramen.

**Figure 6:**
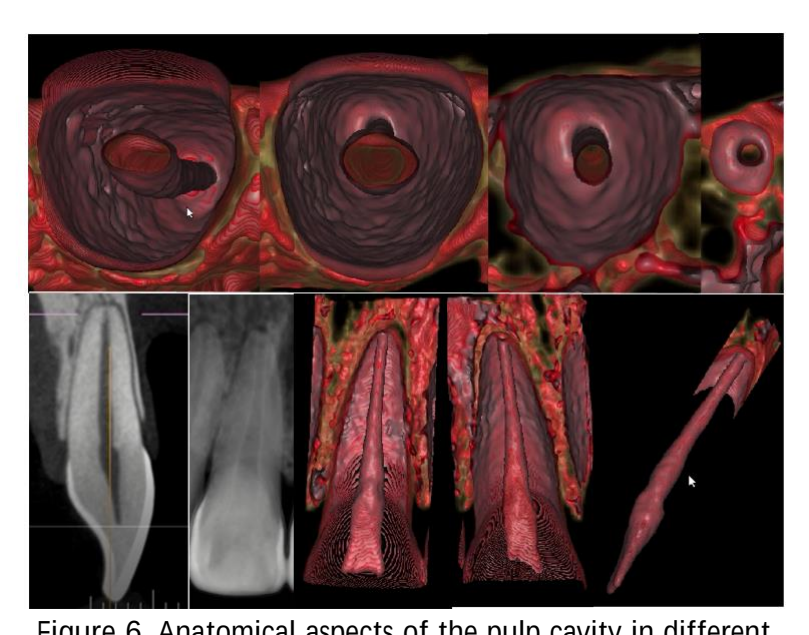
Anatomical aspects of the pulp cavity in different root regions and position of apical foramen in higher magnification and more details of Figure 5.

**Figure 7:**
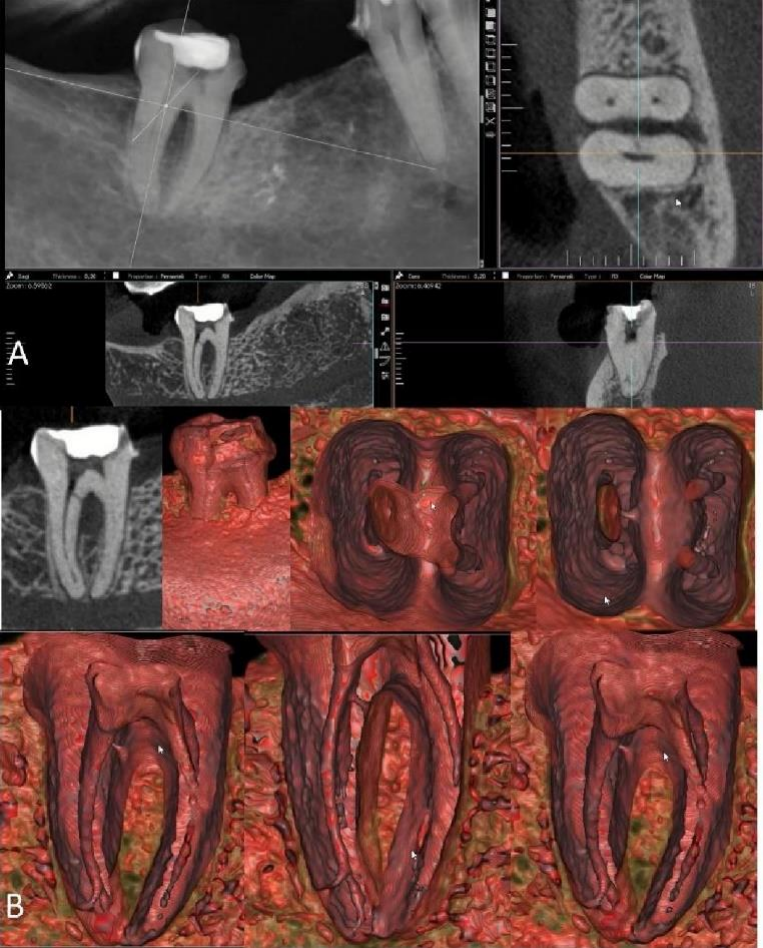
CBCT scan of the mandibular first molar in 2D MPR (A) and 3D cinematic rendering (B). The multidimensional internal navigation showed an open coronal chamber, temporary restorative material, a lateral canal at the coronal level with an extension to the furcation area and an accessory canal at the apical level in the distal root canal. The mesial root canals had normal morphological and anatomical characteristics.

**Figure 8:**
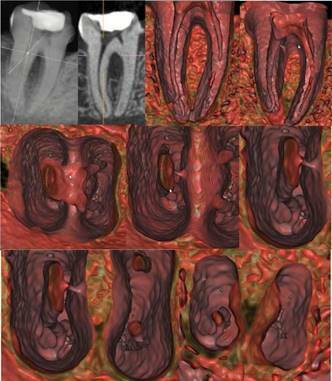
Higher magnification and more details of Figure 7, characterizing the microanatomy of the pulp cavity, accessory canal, and lateral exit of the apical foramen.

A specific 3D volume analysis using a pulp cavity filter and internal scanning was applied to visualize the root canal shape and its anatomical microstructures, such as ramifications and apical foramen. The pulp cavity filter synchronizes 2D CBCT scans for multiplanar reformations (MPR) to 3D mode, for volumetric reconstruction. This filter, when adopting the option of volumetric reconstruction, the developed algorithm leaves the dentin density in transparent mode so that the pulp cavity may be visualized. The algorithm applied performs the suppression (visual) of areas with dentin density. As it identifies the 3D slices, the pulp cavity may be navigated dynamically, ensuring the visualization of the microanatomy of the root canal. The root canals of each tooth were aligned axially, and the sagittal and coronal planes were used to keep the long axis of the tooth parallel to the base to avoid the parallax error. Then, the CBCT scans and the 0.1x0.1-mm axial, sagittal, and coronal sections from the orifice of the coronal chamber to the apical foramen and from the root apex to the coronal region were according to a map-reading strategy. The dynamic navigation was conducted with the CBCT scans in 2D MPR and 3D volumetric reconstruction (Figures 1 to 8). In case 1 (Figure 1,2), the multidimensional visualization of the maxillary right second molar, which had a large coronal restoration, showed the coronal chamber and root canals and revealed the characteristic anatomical shape of each root, the presence of a lateral canal in the palatal root and the position of the apical foramen. In case 2 (Figure 3,4), the inside of the restored mandibular right second premolar was viewed in 3D, showing the nuances of the pulp cavity and the lateral position of the apical foramen terminus. In case 3 (Figure 5,6), the maxillary right central incisor, which had a history of dental trauma, had a large coronal chamber. The multidimensional internal navigation showed the pulp cavity and the central position of the apical foramen. In case 4 (Figure 7,8), the mandibular first molar had an open coronal chamber, temporary restorative material, a lateral canal at the coronal level with an extension to the furcation area and an accessory canal at the apical level in the distal root canal. The mesial root canals had normal morphological and anatomical characteristics.

Using this modeling method for root canal endoscopy, any third of the root canal walls may be viewed, more anatomic information may be obtained, and normal or atypical root canal shapes and the position of an accessories root canal may be identified. Thus, the criteria to evaluate root canal endoscopic examinations may be established according to various parameters, such as the site of accessory root canals, root thirds (apical, middle, and cervical), root surfaces (mesial, distal, buccal, palatal, or lingual), and root association with apical periodontitis. In the clinical cases presented here (Fig. 1,2,3,4,5,6,7,8), the use of the modeling method increased the amount of data generated for the detection of accessory root canals. The use of the volumetric reconstruction filter significantly improved the visualization of the accessory canal in areas of difficult perception.

## Discussion

The contemporary technological revolution has started a new era in the metaverse of Endodontics, a world of virtual operational possibilities that uses an exact replica of the natural structures of the maxillofacial complex. This modeling method for root canal endoscopy using a novel CBCT filter brings an innovative virtual reality and digital perspective to clinical applications, which will be naturally incorporated into the principles of Endodontics. The “metaverse of Endodontics”, a novel endodontic concept, is no longer part of the future and is ready to be incorporated into routine clinical. The immediate application may happen from the incorporation of these new exam resources using digital technologies, such as root canal endoscopy. The use of specific filters of sophisticated CBCT software may provide a more assertive therapeutic decision-making, better predictability of results, and confidence for the professional. The modeling method for root canal endoscopy creates virtual elements of communication about natural tooth structures.

Recently, a pioneering review of the principles of clinical, teaching, and research applications of CBCT cinematic rendering was conducted by Bueno et al. ^10^. The clinical benefits to be added to therapeutic protocols by advances in imaging techniques include CBCT cinematic rendering, which produces 3D images of anatomical structures from more accurate data. A better quality and a greater amount of information may be acquired using cinematic rendering. It is also useful and informative for the differential diagnosis of periapical diseases and the identification of anatomical structures, as well as for planning, clinical decision-making, and teaching.

The use of the high dynamic range resource resulted in highly accurate images, which positively affected the visualization of details used for the analysis of shape and depth. Moreover, image navigation using a specific light source might reveal essential details for a diagnosis and the adoption of a certain treatment plan. The inadequate acquisition and processing of CBCT scans may lead to a misdiagnosis. An improved imaging mode has a strong potential to be used in machine learning and in the development of artificial intelligence tools to aid in diagnosis and treatment planning at the exact time of the operation ^10^.

Several studies have investigated the internal morphology of root canals ^7^ ^,^ ^8^ ^,^ ^9^ ^,^ ^10^ ^,^ ^18^ ^,^ ^20^ ^,^ ^35^ and the potential for new advances including the application of CBCT ^6^ ^,^ ^7^ ^,^ ^8^ ^,^ ^9^ ^,^ ^10^ ^,^ ^35^ ^,^ ^36^. There have also been attempts to find alternatives to a tool that would act as an endoscope inside the root canal. The endoscopes used by otolaryngologists may be adapted for an improved visualization of root apices, especially of those not in the line of direct vision. They may light and identify roots in the sinus if this is necessary for a treatment ^16^.

An endoscopic technique for RCT with an endoscope that combines magnification light, irrigation/suction, and surgical micro-instruments was evaluated using 15 teeth, and findings revealed that endoscopes may improve magnification, irrigation/suction, and shaping during a RCT ^17^. Apical deltas or accessory canals were frequently not identified on radiographs, but the endoscope used in the middle third showed an accessory canal in the maxillary and mandibular first premolars. Endoscopic views show accessory canals better than periapical radiographs ^18^.

Different endoscope optics, that is, pulp endoscopy (PE), canal entrance endos copy (CEE), and root canal endoscopy (RCE) have been evaluated for the visualization of interradicular structures in 20 extracted human mandibular molar teeth ^19^. All pulp chambers were visualized using PE (100%), but only 68.3% root canals were visualized. All entrances were visualized using CEE, and the middle third of the canals was visualized in 85% of the root canals. The semi-flexible RCE endoscope successfully showed 91.6% of the middle third of the canals. Endoscopes may be useful in the identification of root canals even under difficult visual conditions. The combined use of a set of various optics may improve the quality of non-surgical endodontic procedures.

The efficacy of endoscopic visualization to detect the presence and type of isthmuses inside the mesial root canals of mandibular first molars has been recently evaluated and compared with micro-computed tomography (micro-CT) images as a reference ^20^. Thirty-two mesial roots of mandibular first molars with isthmuses were selected on micro-CT scans. The root canals were prepared before endoscopic visualization, and the specimens were mounted in the posterior socket of a dental phantom manikin for endoscopic visualization. Endoscopes with a 15-inch liquid crystal display and thin film transistor monitor with LED backlight at 1024 x 768 resolution showed isthmuses and distinguished the type of isthmuses during the comparison. Micro-CT images of the specimens were used as references. The sensitivity of endoscope to detect isthmuses was also calculated for each isthmus type. In 37.5% of the samples, isthmuses were correctly detected via orthograde endoscopic visualization. Type I isthmuses were significantly more frequently detected than band-shaped isthmuses. Endoscopes had a higher sensitivity to detect type I isthmus than band-shaped isthmus.

Togoe et al. ^21^ analyzed the evolution of two revolutionary methods, using the endoscope and optical coherence tomography, which might improve RCT quality. Research Gate, Science Direct, and PubMed were searched for studies about modern endodontic treatments published between 1990 and 2019 using the following search terms: endoscopy; tooth anatomy; optical coherence tomography; magnification in dental medicine; diagnostics in dental medicine; cariology; restorative dentistry; endodontics; pedodontics; prosthetics; periodontology. One of the advantages of endoscopy is to provide exact 3D images of the endodontic space even beyond root canal curves. An optical coherence tomography is an important tool to establish endodontic diagnoses, as well as to evaluate endodontic treatments.

The method described in this study does not use any endoscopic tool because it is based on the use of a 3D volumetric rendering filter of CBCT software. This filter provides an internal visualization of the root canals during navigation, which simulates the use of an endoscope. In the clinical cases presented here, this computational modeling method increased the amount of information provided because of its dynamic navigation throughout the root canal, showing its anatomical details. Further studies should be conducted to put this method into practice.

The field of imaging studies has developed substantially with the participation of various areas of knowledge and technology. The variety of imaging data that may be acquired and interpreted today has led to the constant incorporation of new computational knowledge into professional practices, all of which require different skills to deal with new sets of data and forms of multidimensional graphic visualization.

The use of this method may potentially provide a complete and comprehensive view of the internal dental anatomy, similarly to an endoscopic examination of the root canal, with a virtual image of CBCT scans acquired from an actual anatomic structure. Many details may be missed in MPR images using CBCT scans. In contrast, virtual reality, as a communication medium that uses photo-realistic images of the maxillofacial structures, may show important details that increase diagnostic accuracy and positively affect clinical decision-making. The high dynamic range resulting from the natural photorealistic quality of the images improves the visualization of the details used for the analysis of shape and depth.

This computational modeling method for root canal endoscopy marks a new era in the world of Endodontics, with a concept of communications and clinical operationalization based on the virtual visualization of the internal anatomy of root canals. It produces an exact replica of the root canal and may positively change the predictability of RCT quality and prognosis, allowing greater confidence for the professional in clinical decision-making.
